# Exaggerated Acute Lung Injury and Impaired Antibacterial Defenses During *Staphylococcus aureus* Infection in Rats with the Metabolic Syndrome

**DOI:** 10.1371/journal.pone.0126906

**Published:** 2015-05-15

**Authors:** Xiaomei Feng, Mervyn Maze, Lauren G. Koch, Steven L. Britton, Judith Hellman

**Affiliations:** 1 Department of Anesthesia and Perioperative Care, University of California, San Francisco, San Francisco, California, United States of America; 2 Department of Anesthesiology, Ruijin Hospital, School of Medicine, Shanghai Jiaotong University, Shanghai, China; 3 Department of Anesthesiology, University of Michigan Medical School, Ann Arbor, Michigan, United States of America; Chinese Academy of Sciences, CHINA

## Abstract

Rats with Metabolic Syndrome (MetaS) have a dysregulated immune response to the aseptic trauma of surgery. We hypothesized that rats with MetaS would have dysregulated inflammation, increased lung injury, and less effective antibacterial defenses during *Staphylococcus (S*.*) aureus* sepsis as compared to rats without MetaS. Low capacity runner (LCR; a model of MetaS) and high capacity runner (HCR) rats were challenged intravenously with *S*. *aureus* bacteria. After 48 h, inflammatory mediators and bacteria were quantified in the blood, bronchoalveolar lavage fluid (BALF), and lung homogenates. Lungs were analyzed histologically. BALF protein and lung wet-dry ratios were quantified to assess for vascular leak. Endpoints were compared in infected LCR vs HCR rats. LCR rats had higher blood and lung *S*. *aureus* counts, as well as higher levels of IL-6 in plasma, lungs and BALF, MIP-2 in plasma and lung, and IL-17A in lungs. Conversely, LCR rats had lower levels of IL-10 in plasma and lungs. Although lactate levels, and liver and renal function tests were similar between groups, LCR rats had higher BALF protein and lung wet-dry ratios, and more pronounced acute lung injury histologically. During *S*. *aureus* bacteremia, as compared with HCR rats, LCR (MetaS) rats have heightened pro-inflammatory responses, accompanied by increased acute lung injury and vascular leak. Notably, despite an augmented pro-inflammatory phenotype, LCR rats have higher bacterial levels in their blood and lungs. The MetaS state may exacerbate lung injury and vascular leak by attenuating the inflammation-resolving response, and by weakening antimicrobial defenses.

## Introduction

Sepsis remains the most common cause of death in non-coronary intensive care units [1–5]. *Staphylococcus* (*S*.) *aureus* is the most common pathogen in gram-positive bacterial sepsis [6–9] and causes infections, ranging from superficial skin infections to severe invasive infections of wounds and organs such as the lung (pneumonia), with full blown septic shock and intractable multiple organ failure [7,10]. *S*. *aureus* infections pose an important health care problem worldwide with expensive medical costs [10–12]. Treatment with antibiotics and the removal of infectious foci are often insufficient to stave off septic shock and multiple organ failure during *S*. *aureus* and other infections. Although multiple organ failure is responsible for half of the mortality of sepsis [13], the mechanisms of sepsis-induced organ failure remain elusive, and little is known about the impact of host factors, including their metabolic profile, on sepsis outcomes.

The Metabolic Syndrome (MetaS) is characterized by insulin resistance (hyperglycemia that can progress to Type 2 Diabetes Mellitus), visceral obesity, hypertension, and dyslipidemia. MetaS is associated with an increased risk of postoperative complications that contribute to substantially higher mortality rates [14–16]. Furthermore, the presence of MetaS enhances the risk of dying in patients with cardiovascular disease [17,18]. Elevated C-reactive protein levels strongly correlate with the number of the components of MetaS that the patient exhibits and are thought to reflect a low-grade pro-inflammatory state [19].

To better understand the pathophysiology leading to the increase in complications in the setting of MetaS, investigators have developed rodent models. Genome-wide association studies suggest that MetaS is a polygenic disorder [20–22]. Therefore we were attracted to animal reagents that were developed from a founder population of genetically heterogeneous rats by applying divergent artificial selection for intrinsic low and high endurance running capacity [22]. More than thirty generations of selection produced lines of low capacity runners (LCRs) and high capacity runners (HCRs) that differ markedly in treadmill running capacity [23]. Compared with HCR rats, LCR rats exhibit multiple features of MetaS, including hyperlipidemia, hypertension, high fasting glucose, elevated C-reactive protein, and visceral adiposity [24]. While exploring the mechanisms of an exaggerated and persistent form of postoperative cognitive decline in the setting of MetaS [25], we discovered that LCR rats are defective in several steps of postoperative inflammation resolution [26].

Numerous host factors modulate immune responses, leading to substantially different outcomes between individuals with similar infections. Diabetes and obesity each increase the risk of both developing infections and of having worse early outcomes during serious infections [27–34]. Our prior studies, showing that LCR rats are defective in postoperative resolution of inflammation [35], suggested to us that similar immune system dysfunction might contribute to the complications of sepsis. Of note, while altered responsiveness to viral infection and its treatment in the setting of MetaS is well chronicled, little is known about how MetaS modulates bacterial sepsis [36–38].

The goal of these studies was to test the hypothesis that during *S*. *aureus* sepsis, LCR rats have hyperinflammatory responses and altered bacterial clearance as compared to HCR rats.

## Materials and Methods

### Ethics statement

This study was carried out in strict accordance with the recommendations in the Guide for the Care and Use of Laboratory Animals of the National Institutes of Health. The protocol was approved by the Institutional Animal Care and Use Committee (IACUC) of the University of California, San Francisco (Protocol Number: AN090565). All procedures were performed under ketamine and xylazine anesthesia, and all efforts were made to minimize suffering.

### Animals

The development of rats selected to be either LCR or HCR is described in detail elsewhere [22,23]. In the present investigation, female HCR and LCR rats (generation 31) were housed under standard laboratory temperature and humidity conditions in which the light and dark cycles were 12 h each. Rats were tested for running capacity at the University of Michigan at 11 weeks of age and shipped to the University of California, San Francisco at 6 months of age. Similar aged female Sprague-Dawley rats (Jackson Laboratories) were used for pilot studies to optimize dosing and endpoint analyses in the *S*. *aureus* primary bacteremia model.

### Bacterial strains and growth conditions

*S*. *aureus* Newman strain is a methicillin-sensitive strain that was initially isolated from an infected patient [39]. *S*. *aureus* Newman can induce severe infections with organ injury and lethality in rodents, and is often used for early initial proof of concept studies, such as this one, because it is methicillin-sensitive and can be used safely in laboratory animals. *S*. *aureus* was cultured at 37°C in Lysogeny Broth (LB) medium. Bacterial stocks were kept at -80°C in LB medium supplemented with 20% (vol/vol) of glycerol. Overnight cultures of *S*. *aureus* were re-inoculated into fresh LB and grown to log phase. The bacteria were then harvested by centrifugation, washed, and resuspended in saline at the appropriate concentration for the final inoculum size. The inoculum size, in colony forming units (CFU), was confirmed by quantifying colony counts on blood agar plates.

### Induction of *S*. *aureus* primary bacteremia

Rats were anesthetized with ketamine hydrochloride (80 mg/kg IP) and xylazine (8 mg/kg IP), placed in the supine position, and *S*. *aureus* was administered intravenously (IV) in a volume of 1 ml sterile normal saline via a sterile cannula inserted into the lateral tail vein. Pilot dose response studies using Sprague-Dawley rats (n = 4/group) were used to establish a dose of *S*. *aureus* that reproducibly causes sustained bacteremia and the induction of inflammatory mediators without causing mortality in the 48 h timeframe of the study. For the definitive experiments, both HCR and LCR rats were challenged IV with an identical inoculum of 5x10^7^ CFU/kg dose based on the mean body weight of HCR rats (n = 7/group). The rats were returned to their cages and monitored daily for signs of inability to right themselves, respiratory distress (e.g., irregular breathing, wheezing), or ruffled fur. We verified that control HCR and LCR rats treated with sterile saline (n = 3/group) had background levels of cytokines comparable to levels in untreated rats. All control rats that received saline had sterile cultures of their blood, BALF and lung homogenates.

### Collection of blood, BALF and lung tissue, quantification of bacterial counts, cytokines, and lung edema

At 48 h, the rats were sacrificed under deep anesthesia provided by ketamine/xylazine. Blood samples were collected immediately from the inferior vena cava in vacutainer plasma tubes. In a separate set of rats, 2 ml bronchoalveolar lavage fluid (BALF) was collected *via* tubes place in the trachea. All subsequent steps were carried out at 0–4°C. Following rinsing, the right lungs were removed, weighed aseptically, and homogenized in 1 ml cold PBS for 1 min. The homogenizer was sterilized between samples to avoid cross-contamination of bacteria between different samples. Blood, BALF, and the lung homogenates were processed immediately after collection. Serial 10-fold dilutions were performed, and 100 microliters of appropriate dilutions were spread on blood agar plates in triplicate. Plates were then incubated for 48 h at 37°C, and colonies were counted on plates that contained between 5–300 CFU. CFU/ml of lung homogenates, BALF, or blood were calculated by multiplying the number of colonies on the plate by 10 and then by the dilution factor used for the plate. For lung homogenates, CFU/mg was further calculated based on the weight of the organ prior to homogenization.

Total protein concentration was measured in the BALF fluid (Pierce BCA Protein Assay Kit, Thermo Scientific, Rockford, Illinois). Lung wet-dry weight ratios were quantified using the left lungs. Excess fluid was blotted from the lung. The wet weight was measured immediately, and then the lungs were dried at 80°C for 72 hours and weighed again to determine the dry weights.

ELISAs were performed to quantify IL-6, MIP-2, IL-10 (all from R&D Systems), and IL-17A (eBiosciences) in plasmas, BALF, and lung homogenates.

### Markers of liver and kidney function, electrolytes, CO_2_, and lactate

Plasma levels of liver function (aspartate aminotransferase [AST] and alanine aminotransferase [ALT]), kidney function (blood urea nitrogen [BUN] and creatinine [Cr]), electrolytes (sodium [Na^+^], potassium [K^+^], and Chloride [Cl^-^]), CO_2_, and lactate were quantified by the clinical laboratory of San Francisco General Hospital.

### Histology

The lungs of rats were fixed in 4% paraformaldehyde and paraffin-embedded. Sections of 5 micron thickness were cut using a microtome and stained with hemotoxylin and eosin (H&E).

### Statistical analysis

Data in the Figures are expressed as median and interquartile range. Statistical analyses for all data were performed with 2-tailed nonparametric Mann-Whitney U tests. P values of < 0.05 were considered statistically significant. Analyses were done using GraphPad Prism 6 software, San Diego, CA. Comparisons were made between *S*. *aureus*-infected LCR versus *S*. *aureus*-infected HCR rats. Group sizes of n = 7/group were chosen based on a power analysis setting a statistical significance of p < 0.05, a power of 80%, an anticipated treatment difference (infected HCR versus infected LCR) of ≥10%, and a standard deviation of 10%. This group size was also found to be appropriate in our previous studies addressing functional changes in this Metabolic Syndrome model [25] and inflammatory responses to provocative stimuli in this Metabolic Syndrome model [26]. The values for levels of cytokines, protein in BALF, or lung weight-dry ratios were calculated by subtracting the background levels of these markers in saline-treated rats from the levels in rats infected with *S*. *aureus* rats (i.e., cytokine value shown on Y axis for LCR rats = cytokine level of LCR with *S*. *aureus* infection—cytokine level of LCR rat treated with sterile saline).

## Results

### Pilot dose-response study in Sprague-Dawley rats

We established the appropriate *S*. *aureus* dose using adult female Sprague-Dawley rats. With the goals of inducing moderate inflammation as well as the hematogenous seeding of *S*. *aureus* in the lungs, but without causing mortality, we challenged rats IV with 5×10^6^ − 5×10^7^ CFU/kg (n = 4/group). Two days after IV challenge with 5×10^7^ CFU/kg *S*. *aureus*, all rats were alive, the mean bacterial count was 938 CFU/g lung tissue, and there was robust induction of IL-6 in lung homogenates (data not shown). Therefore, we chose to proceed with our study in LCR versus HCR rats using a *S*. *aureus* dose of 5×10^7^ CFU/kg based on the mean weight of the HCR rats.

### Weight of LCR and HCR rats before and 48 h after *S*. *aureus*

**Fig 1:**
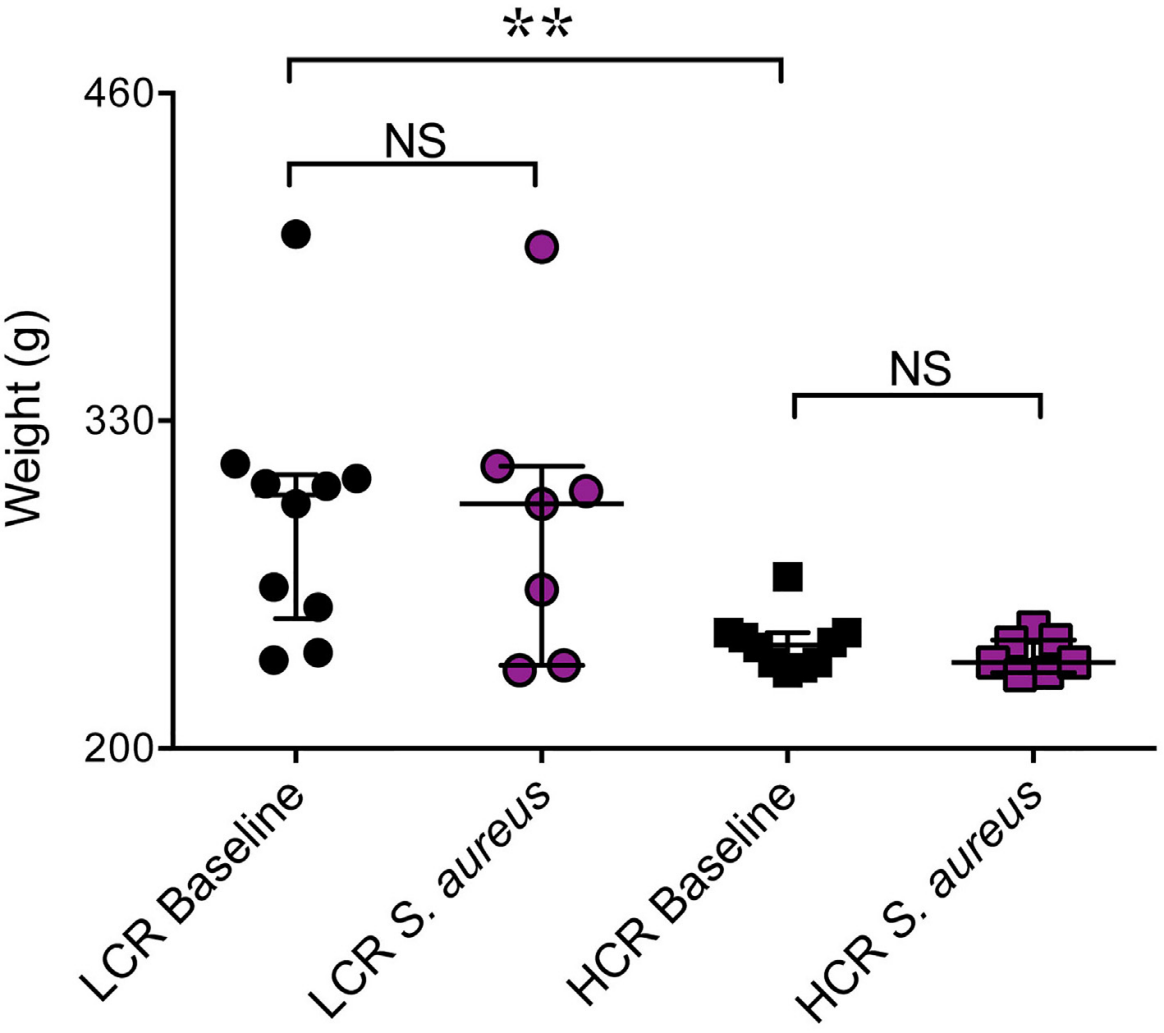
Body weight of the HCR and LCR rats before and during infection with *S*. *aureus*. LCR rats (Circles) and HCR rats (Squares) were weighed before (n = 10/group) and 48 h after they were challenged IV with *S*. *aureus* (n = 7/group, purple filling). LCR rats had significantly higher body weights at baseline than HCR rats (**p = 0.005). There were no significant differences between weights at baseline and after 48 h of infection for either LCR or HCR (*p > 0.05, LCR baseline versus LCR-*S*. *aureus* and HCR baseline versus HCR-*S*. *aureus*, Mann Whitney U tests). Results are expressed as median with interquartile range. HCR = high capacity runner; LCR = low capacity runner.

At baseline, prior to infection with *S*. *aureus*, the mean weight of HCR rats (242 ± 11 g) was significantly lower than that of LCR rats (292 ± 49 g), (Fig 1, p = 0.005). However, there were no significant differences in the weights of LCR rats prior to *vs* 48 h after challenge with *S*. *aureus* (Fig 1, p > 0.05). Similarly, there were not significant differences in the weights of HCR rats prior to *vs* 48 h after *S*. *aureus* challenge (Fig 1, p > 0.05).

### Bacterial levels are higher in the blood, BALF, and lung homogenates of infected LCR rats versus infected HCR rats

**Fig 2:**
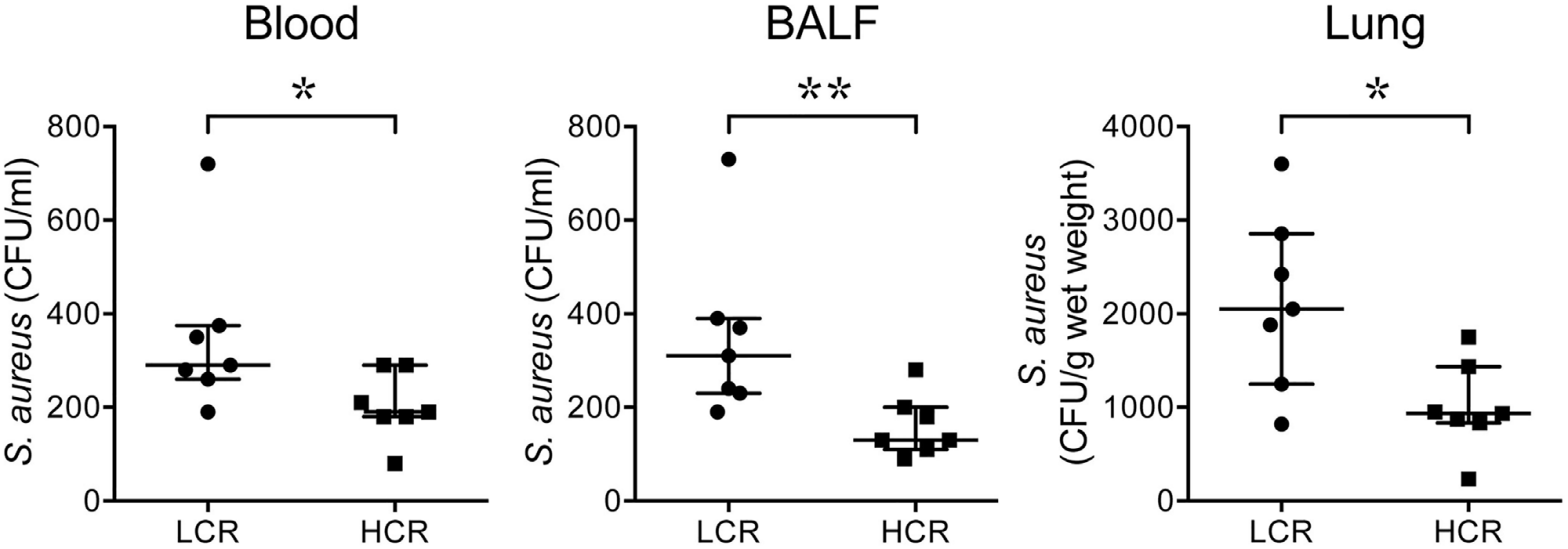
Bacterial levels in rats with *S*. *aureus* bacteremia. LCR rats (Circles) and HCR rats (Squares) were challenged IV with *S*. *aureus* (n = 7/group). 48 h later levels of *S*. *aureus* were significantly higher in the blood, BALF, and lung homogenates of LCR as compared with HCR rats (*p = 0.041, **p = 0.0006, and *p = 0.038, respectively, Mann Whitney U tests). Results are expressed as median with interquartile range. HCR = high capacity runner; LCR = low capacity runner; BALF = bronchoalveolar lavage fluid; CFU = colony-forming units.

Challenge with *S*. *aureus* produced a robust bacterial load in the circulation and in lungs (BALF and lung homogenates) at 48 h (Fig 2). HCR and LCR rats that received saline (carrier for the *S*. *aureus*) had no CFU’s in their blood, BALF or lung homogenates (n = 3/group). Levels of *S*. *aureus* (as assessed by CFUs) were significantly higher in the blood, BALF, and lung homogenates of LCR versus HCR rats (n = 7/group, p = 0.041, 0.006, and 0.038, respectively).

### Cytokine levels in the blood, BALF, and lung homogenates of infected LCR rats versus infected HCR rats

**Fig 3:**
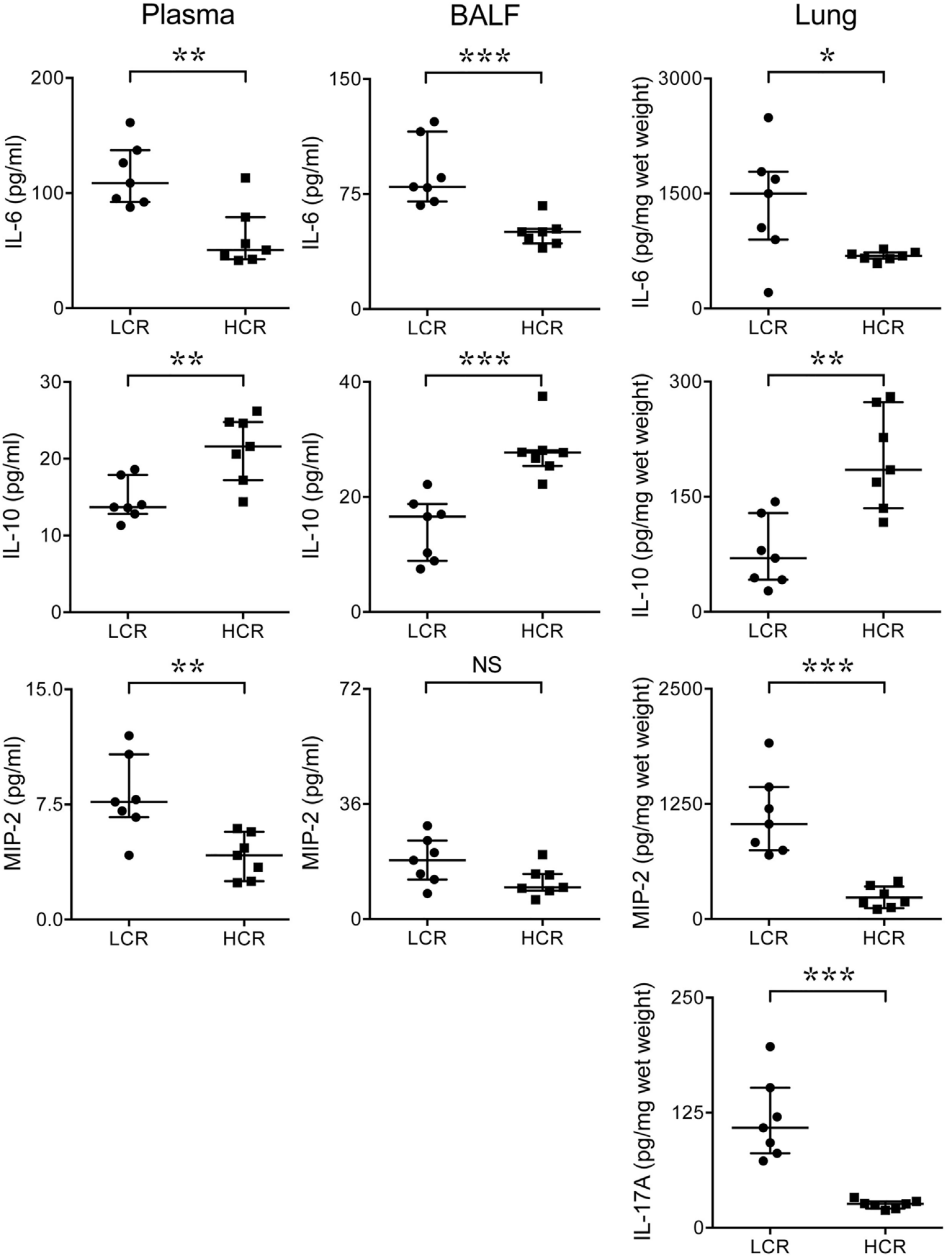
Systemic and lung cytokine and chemokine levels in LCR and HCR rats infected with *S*. *aureus*. LCR rats (Circles) and HCR rats (Squares) were challenged IV with *S*. *aureus* (n = 7/group). Levels of IL-6, IL-10, MIP-2, and 1L-17A were quantified in the serum, BALF, and lung homogenates 48 h after challenge with *S*. *aureus*. Compared with infected HCR rats, infected LCR rats had higher levels of IL-6 in plasmas, BALF and lung homogenates (**p = 0.007, **p = 0.0006, and *p = 0.0262, respectively), higher levels of MIP-2 in plasmas and lung homogenates (**p = 0.004 and **p = 0.0006, respectively), and higher levels of IL-17A in lung homogenates (**p = 0.0006). In contrast, compared with infected HCR rats, infected LCR rats had lower levels of IL-10 in plasmas, BALF and lung homogenates (**p = 0.007, ***p = 0.0006, and **p = 0.0041, respectively). The figures show levels of cytokines after subtracting out the values measured in control rats treated with sterile saline from the values measured in rats treated with *S*. *aureus* (i.e.: cytokine value shown on Y axis for LCR rats = cytokine level of LCR with *S*. *aureus* infection—cytokine level of LCR rat treated with sterile saline, similarly for HCR rats). Data were analyzed using Mann Whitney U tests; results are expressed as median with interquartile range. HCR = high capacity runner; LCR = low capacity runner; BALF = bronchoalveolar lavage fluid; IL = interleukin; MIP = macrophage inflammatory protein.

Cytokines and chemokines, including IL-6, IL-10, IL-17A, and MIP-2 were induced systemically and in the lungs of rats with *S*. *aureus* bacteremia. IL-6 levels were higher in the blood, BALF, and the lung of infected LCR versus infected HCR rats (Fig 3, p = 0.0070, 0.0006, and 0.0262, respectively). In contrast to IL-6, levels of the anti-inflammatory cytokine, IL-10, which plays an important role in resolving inflammation, were significantly lower in the blood, BALF, and lung homogenates of infected LCR versus HCR rats (Fig 3, p = 0.007, 0.0006, and 0.0041, respectively). Levels of MIP-2 were significantly higher in the blood and lungs of infected LCR rats versus HCR rats (Fig 3, p = 0.004, 0.001), but not in the BALF of LCR rats versus HCR rats (Fig 3, p = 0.053). IL-17A was significantly higher in lung homogenates of infected LCR versus infected HCR rats (p = 0.001), but was not detectable in plasmas or BALF or either HCR or LCR rats (data not shown).

### Markers of liver and kidney function, electrolytes, CO_2_, and lactate

There were no significant differences in levels of the liver enzymes, AST and ALT, parameters of kidney function (BUN and Cr), electrolytes (Na^+^, K^+^, and Cl^-^), lactate, or CO_2_ between infected HCR and LCR rats (Data not shown).

### Lung vascular edema and histology in LCR and HCR rats infected with *S*. *aureus*

**Fig 4:**
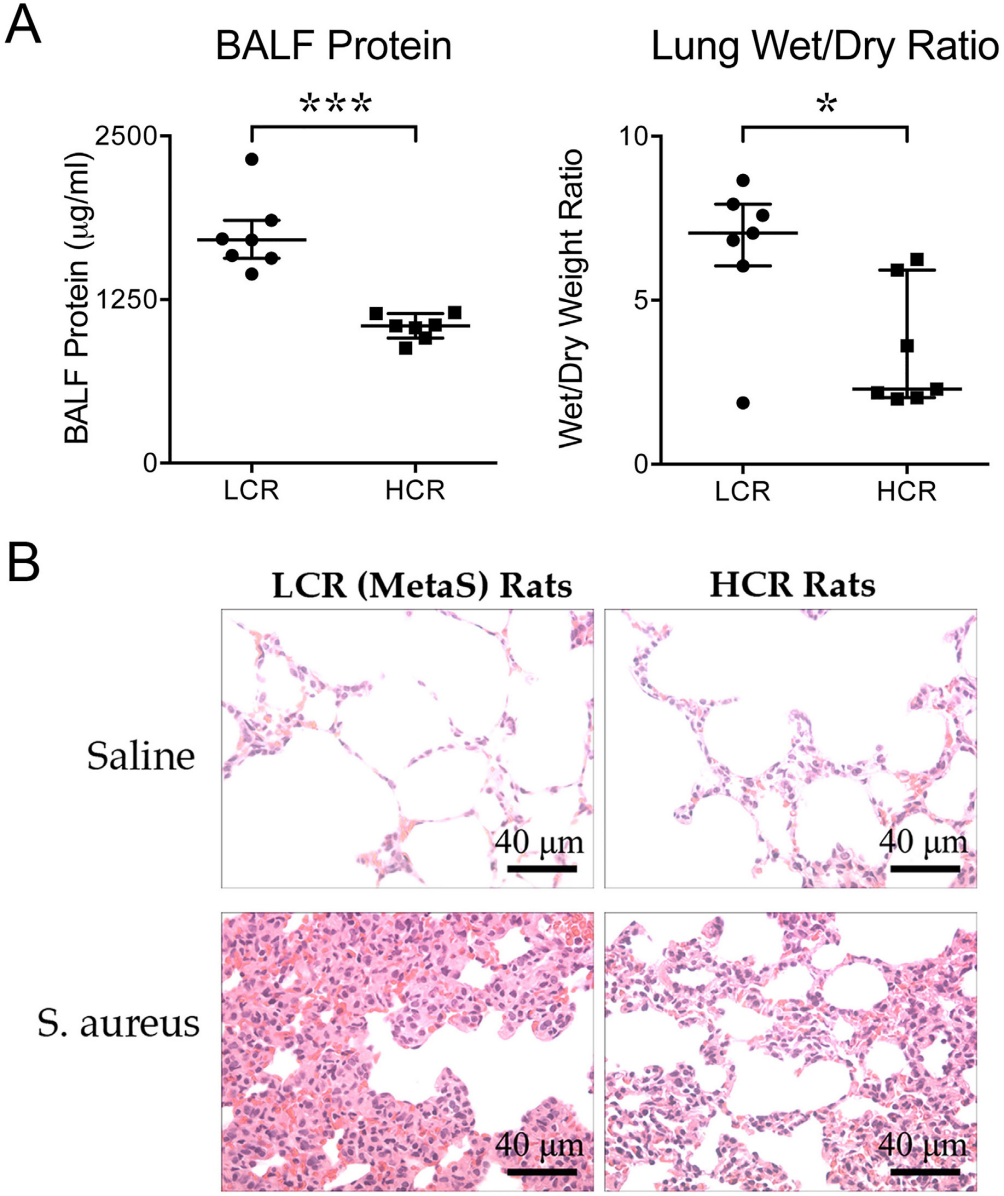
Lung permeability, edema and histology in LCR and HCR rats infected with *S*. *aureus*. Protein level in the BALF, lung wet-dry weight ratios, and lung histology were assessed 48 h after LCR and HCR rats were challenged IV with *S*. *aureus* (n = 7/group). (A) Compared with infected HCR rats (Squares), infected LCR rats (Circles) had higher levels of BALF protein and higher lung wet-dry weight ratios (***p = 0.0006 and *p = 0.0379, respectively, Mann Whitney U tests). Results are expressed as median with interquartile range. The values shown in the figures represent the BALF protein and lung wet-dry ratios after subtracting out the values in control rats treated with sterile saline from the levels measured in rats treated with *S*. *aureus* (i.e.: BALF protein or lung wet-dry ratio value shown on Y axis for LCR rats = BALF protein or lung wet-dry ratio of LCR with *S*. *aureus* infection—BALF protein or lung wet-dry ratio of LCR rat treated with sterile saline, similarly for HCR rats). (B) H & E staining of lung sections demonstrated augmentation of inflammatory cell influx, blood, edema, and thickening of the lung interstitium in LCR rats as compared with HCR rats (40-fold magnification, scale bar = 40 μm). HCR = high capacity runner; LCR = low capacity runner. BALF = bronchoalveolar lavage fluid.

*S*. *aureus* infection caused increased lung vascular permeability to protein and increased lung wet-dry weight ratios, both indicators of pulmonary edema. The range of BALF protein and lung wet-dry weight ratios were consistent with those reported in the literature in acute lung injury in rats [40]. BALF protein levels were substantially higher in infected LCR rats as compared with HCR rats (Fig 4A, left panel; p = 0.0006). Similarly, the lung wet-dry weight ratio was higher in LCR rats versus HCR rats (Fig 4A, right panel; p = 0.0379). H&E staining of lung sections (Fig 4B) demonstrated an inflammatory cell influx, edema, extravascular blood cells, and thickening of the interstitium in the lungs of *S*. *aureus*-infected rats, which were all substantially more pronounced in LCR versus HCR rats with *S*. *aureus* infection.

## Discussion

These studies indicate that, as compared with HCR rats, LCR (MetaS) rats with *S*. *aureus* bacteremic sepsis have higher systemic and lung levels of bacteria (Fig 2) and pro-inflammatory cytokines and chemokines (Fig 3), and reduced levels of the anti-inflammatory cytokine, IL-10 (Fig 3) after 48 h of infection. Consistent with the cytokine and chemokine profile and higher bacterial levels in LCR rats, *S*. *aureus* infection also caused more profound vascular leak, as evidenced by increased BALF protein levels and increased lung wet-dry weight ratios (Fig 4A), and well as more pronounced histologic evidence of lung inflammation and injury (Fig 4B) in LCR *vs* HCR rats. There were no differences in levels of lactate or liver enzymes, or parameters of renal function in HCR *vs* LCR rats. However, at the 48 h collection time, infected HCR and LCR rats had very low levels of these markers of organ dysfunction and perfusion, which suggests that they not yet developed severe shock and multiple organ failure. Thus the study does not rule out a role for the MetaS in the later development of shock and multiple organ failure. Taken together, our novel findings support an important role for the Metabolic Syndrome in exacerbating a sustained pro-inflammatory response, early lung injury, and lung vascular permeability, and in reducing the host’s defense against *S*. *aureus* infection. The higher levels of bacteria in the blood and lungs of LCR rats is even more notable when one considers that the dosing of *S*. *aureus* was based on the mean weight of the HCR rats (242 g) which was significantly lower than that of the LCR rats (292 g). Thus the *S*. *aureus* dose was, in fact, lower on a per weight basis in LCR rats (4.1x10^7^ CFU/kg) than in HCR rats (5x10^7^ CFU/kg).

We chose to use LCR rats as a surrogate model of MetaS, rather than isolated genetically-manipulated animals such as the leptin-deficient *ob/ob* and leptin-resistant *db/db* mice [41], because MetaS is likely a polygenic rather than monogenic disorder. This is suggested by the multiplicity of metabolic derangement encountered in humans with MetaS. The LCR and HCR rat lines were generated by two-way (divergent) artificial selective breeding for low and high intrinsic (i.e., untrained) capacity for treadmill running performance. The selection was initiated using the genetically heterogenous stock of N/NIH rats as the founder population [23]; at generation 28 the lines differed by over 8-fold in running performance [42]. LCR rats display standard features of MetaS, including, elevated cholesterol, blood pressure, triacylglycerols, fasting glucose, insulin, visceral adiposity, and body weight [24]. The aggregation of disease risk factors in the LCR rats may be at least partly explained by diminished energy metabolism and defective mitochondrial function relative to the HCR rats [43].

Because acute inflammation caused by sterile and infectious processes share many immunologic features, our earlier identification of alterations in the inflammation resolution following aseptic trauma in a model of MetaS are pertinent to our current study. These alterations in the innate immune response, which include resistance to the inflammation-resolving cholinergic actions of α7nAChR agonists, fewer circulating regulatory T cells to orchestrate anti-inflammatory responses, and eicosanoid products that are pro-inflammatory *in lieu* of pro-resolving [26], are likely responsible for the exaggerated inflammation and concurrent impaired bacterial clearance in response to *S*. *aureus* bacteremic sepsis in rats. While inflammation is required to control and clear microorganisms during sepsis, under some circumstances, an abnormal inflammatory state characterized by high levels of pro-inflammatory cytokines, but also immunosuppression, prevails in sepsis and is associated with high lethality [44].

In our earlier studies pursuing the mechanisms for postoperative cognitive decline we established that inflammation-resolving mechanisms involving neural (cholinergic) and humoral pathways [45] are launched in tandem with pro-inflammatory cytokine secretion; a dampening of these resolving mechanisms is implicated in exaggerating postoperative cognitive decline [46]. In our current report, in which we explore the lung as the target organ for a dysregulated immune system, we observed similar changes as previously seen in our surgical model. These included (1) a pro-inflammatory cytokine panel, (2) histological evidence of a sustained pro-inflammatory response associated with a profound leukocyte infiltration, (3) increased expression of the chemokine MIP-2, a major participant in leukocyte mobilization from the bone marrow and migration to sites of acute inflammation, and (4) increased expression of IL-17A. The last-mentioned, produced by helper T lymphocytes, is involved in inducing tissue chemokine production and is believed to contribute to tissue damage [47,48]. In the setting of a dysregulated immune system, whether it is engaged by trauma, infection, or other provocative factors, patients with MetaS may poorly tolerate diseases such as cancer and infection, which have a pivotal immune component. Indeed, there is growing evidence suggesting that the failure to resolve inflammation contributes to poor outcomes in acute inflammatory disorders such as sepsis [49–51]. Our results that show increased systemic and lung pro-inflammatory cytokines/chemokines, and lower levels of the anti-inflammatory cytokine, IL-10, in LCR versus HCR rats suggest that MetaS may contribute to this failure to shift to a pro-resolving phase.

Our study may have direct relevance to humans with sepsis and multiple organ failure caused not only by *S*. *aureus*, but also by other bacteria. In humans, MetaS is associated with increased rates of infection and with worse acute outcomes of infections [27–34]. Surprisingly, however, studies suggest that obesity, a component of the MetaS, does not worsen, and may positively impact long-term outcomes of human sepsis [52–54]. The possibility that obesity may serve a protective role in sepsis is supported by a recent report that obese patients have higher one year survival rates than nonobese patients [55]. However, studies also suggest that obese patients have longer ICU length of stay as well as higher rates of MODS, a complication of acute inflammatory critical illness [53,56]. Our study focused only on the acute stages of *S*. *aureus* sepsis, and the results neither support nor refute the concept that obesity may improve long-term outcomes of sepsis. Furthermore, we cannot extrapolate from our study the specific contribution that obesity had on the acute outcomes because LCR rats have multiple derangements in addition to obesity, including insulin resistance, hyperlipidemia, and hypertension. Ultimately, genetically modified mice rather than the polygenic rat model of MetaS should be helpful in defining the independent effects of the elements of MetaS, including obesity, on short- and long-term outcomes.

Sepsis and critical illness are associated with cellular immune dysfunction, which increases susceptibility to infections, and may potentiate organ injury due to lack of appropriate resolution of acute inflammation [44]. Our data suggest that MetaS may exacerbate inflammation as well as acute lung injury through a failure to resolve the pro-inflammatory response. While the identification of the specific mediators involved in the worse outcomes of septic rats with MetaS (LCR) is beyond the scope of the current manuscript, we speculate that differences in specific pro-resolving mediators (SPMs) in the LCR versus HCR rats may contribute to the different outcomes in LCR and HCR rats. A number of recent studies support an important role for lipid-derived SPMs, such as resolvins and lipoxins in resolving inflammation [50]. Notably, the available data suggest that pathways involved in resolution of inflammation also promote antibacterial defenses [57,58]. Consistent with reports on resolvins and other lipid mediators in sepsis, our data suggest that despite promoting a pro-inflammatory state, MetaS also leads to a state of immune dysfunction with reduced bacterial clearance.

Severe *S*. *aureus* pneumonia and bacteremia are associated with high mortality rates, and cause substantial morbidity in survivors [10–12,59]. The development of tissue foci of infections in patients with *S*. *aureus* bacteremia, and conversely of bacteremia in patients with localized *S*. *aureus* infections, portends worse outcomes [6–9]. Current treatment options for sepsis caused by *S*. *aureus* and other microorganisms are limited to antibiotics, removal of infectious sources, and the supportive care of failing organs. Strategies that globally interfere with pro-inflammatory mediators or receptors have not been successful in clinical trials, and the failures of several recent Phase 3 human sepsis trials provide the impetus to identify novel therapeutic targets [5,60–62]. The available evidence suggests that the Metabolic Syndrome, which is prevalent in humans in developed countries, promotes more profound dysregulation of inflammatory responses and is associated with worse acute and subacute outcomes in critically ill patients. Our study is in line with this paradigm, and suggests that MetaS reduces the host’s ability to clear bacteria from the bloodstream and facilitates bacterial seeding of the lung during hematogenous *S*. *aureus* infection. The identification of factors that differ between individuals with and without the MetaS may provide novel mediators or pathways to target therapeutically.

## Supporting Information

S1 ARRIVE Checklist (DOCX)
