# Supplementary material for: Exaggerated Acute Lung Injury and Impaired Antibacterial Defenses During Staphylococcus aureus Infection in Rats with the Metabolic Syndrome
Source: PLoS One. 2015 May 15;10(5):e0126906. doi: 10.1371/journal.pone.0126906 (PMC4433232; doi:10.1371/journal.pone.0126906)

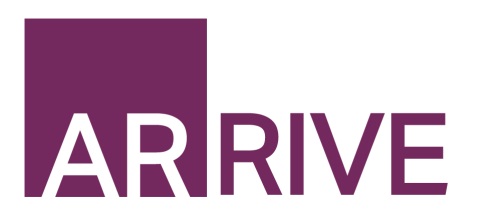


The ARRIVE Guidelines Checklist

Animal Research: Reporting In Vivo Experiments

Carol Kilkenny^1^, William J Browne^2^, Innes C Cuthill^3^, Michael Emerson^4^ and Douglas G Altman^5^

*^1^The National Centre for the Replacement, Refinement and Reduction of Animals in Research, London, UK, ^2^School of Veterinary Science, University of Bristol, Bristol, UK, ^3^School of Biological Sciences, University of Bristol, Bristol, UK, ^4^National Heart and Lung Institute, Imperial College London, UK, ^5^Centre for Statistics in Medicine, University of Oxford, Oxford, UK.*

|  | | ITEM | RECOMMENDATION | Section/ Paragraph |
| --- | --- | --- | --- | --- |
| 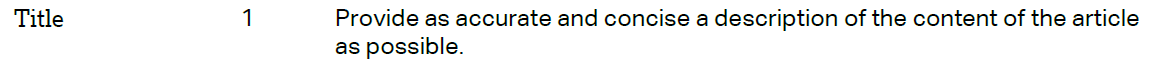 | | | Title |  |
| 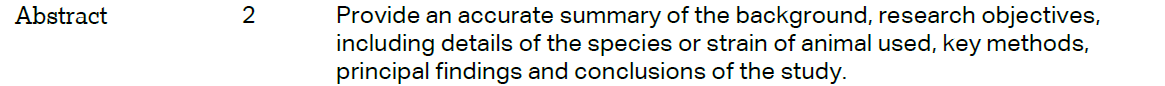 | | | Abstract |  |
| INTRODUCTION | | |  |  |
| 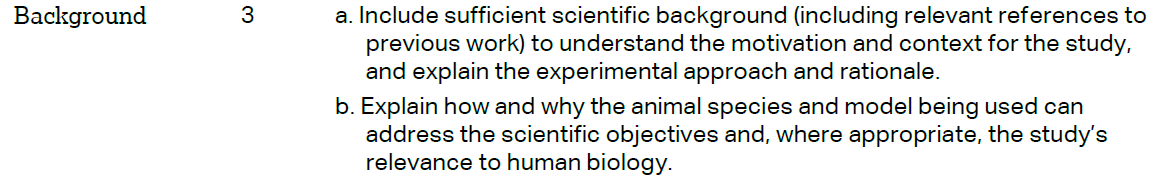 | | | Paragraphs 1-4 |  |
| 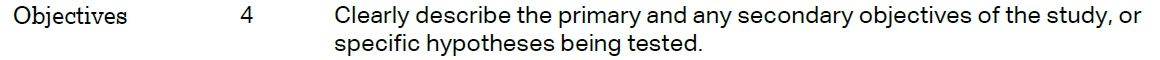 | | | Paragraph 5 |  |
| METHODS | | |  |  |
| 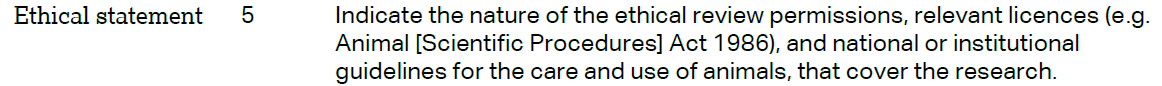 | | | Paragraph 1 |  |
| 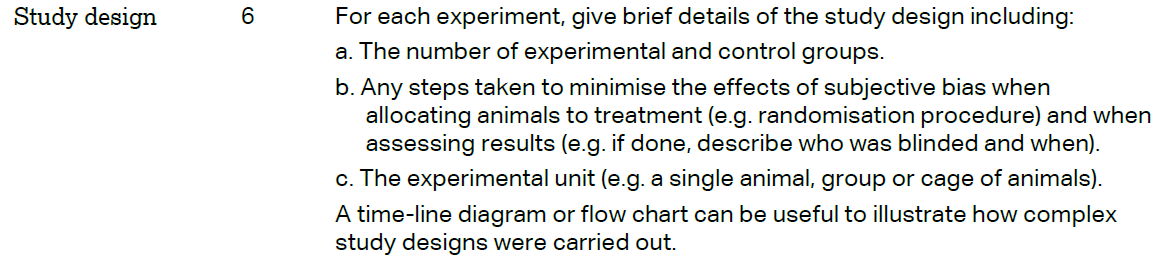 | | | Paragraphs 4, 10 |  |
| 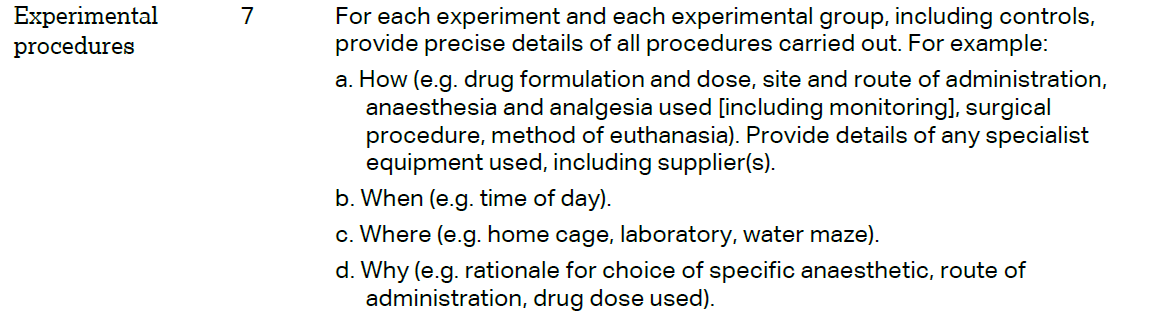 | | | Paragraphs 3-9 |  |
| 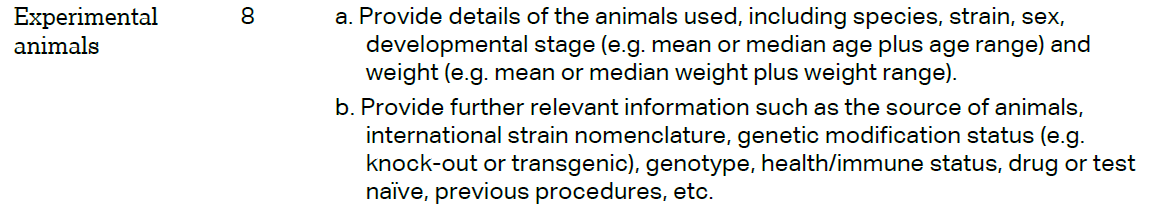 | | | Paragraph 2 |  |

The ARRIVE guidelines. Originally published in *PLoS Biology*, June 2010^1^

| 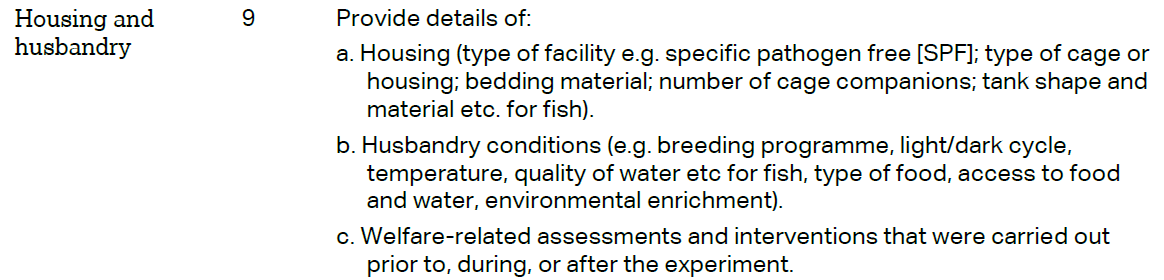 | Paragraph 1, 2 | |
| --- | --- | --- |
| 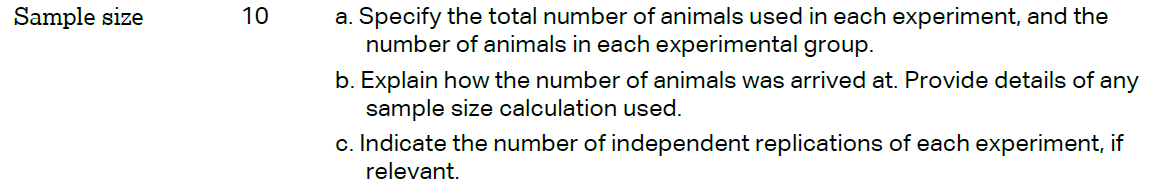 | Paragraph 10 | |
| 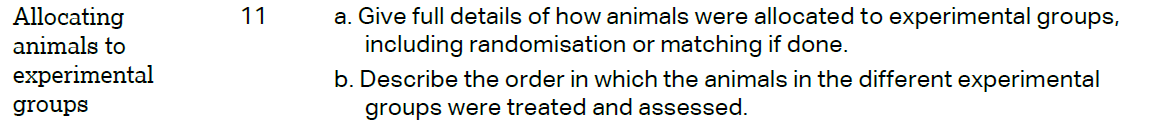 | Paragraph 10 | |
| 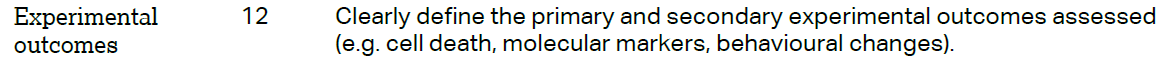 | Paragraphs 6-9 | |
| 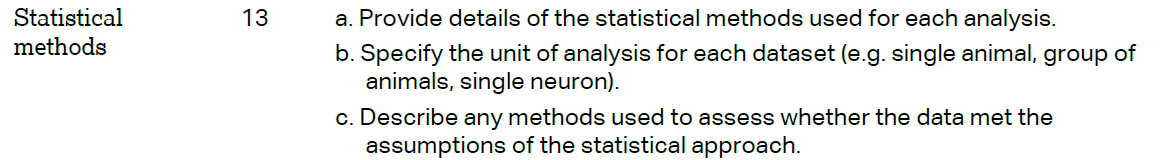 | Paragraph 10, All Figure Legends | |
| RESULTS |  | |
| 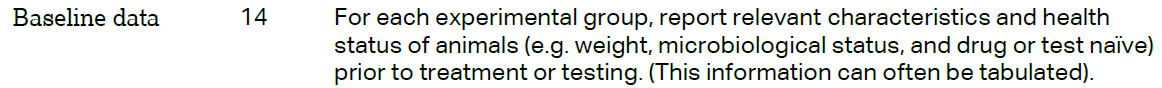 | Paragraphs 2, 6 | |
| 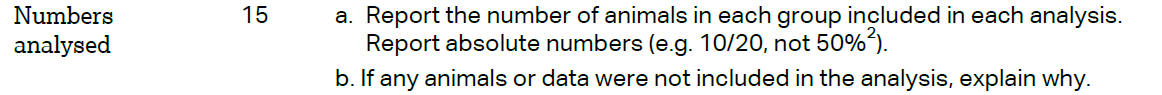 | Paragraphs 1, 2, 4, 6, 9 | |
| 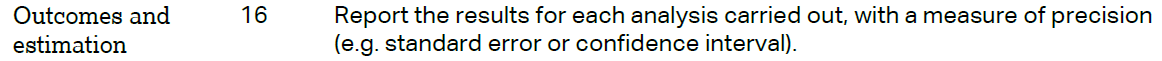 | Paragraphs 2, 4, 6, 9 Figures 1, 2, 3, 4 | |
| 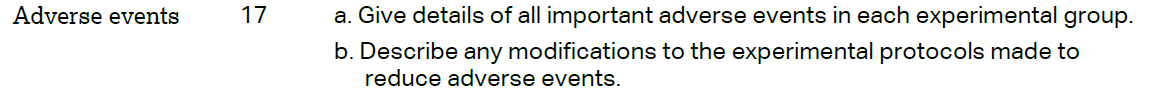 | Not applicable | |
| DISCUSSION |  | |
| 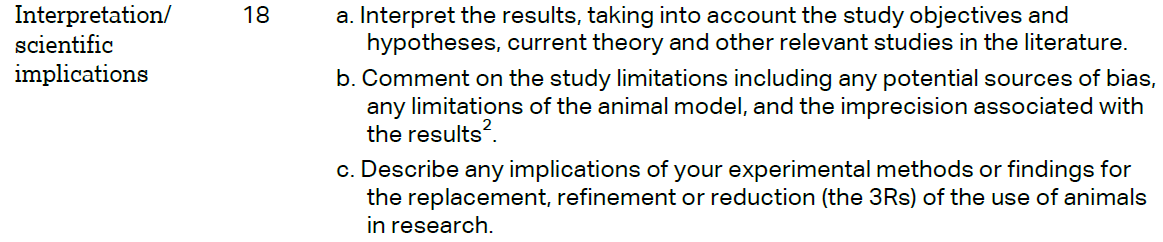 | Paragraphs 1, 2, 4, 5, 6 | |
| 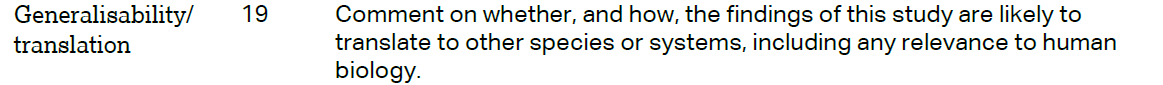 | 5, 6, 7 | |
| 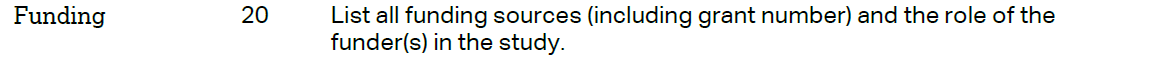 | |  |


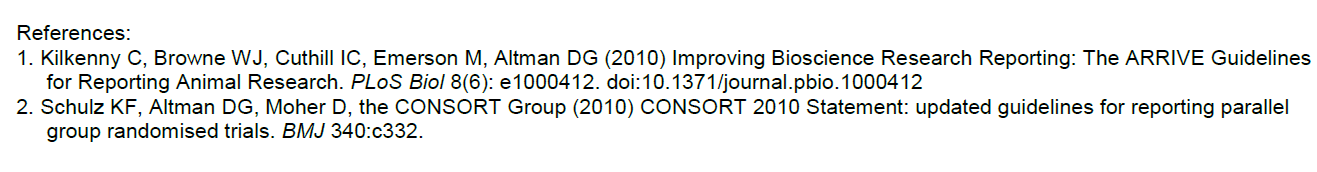

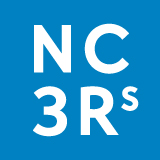

Supplement: S1 ARRIVE Checklist — (DOCX) [file pone.0126906.s001.docx]
